# Two-year clinical performance of indirect resin composite restorations in endodontically treated teeth with different cavity preparation designs: a randomized clinical trial

**DOI:** 10.1186/s12903-024-04725-5

**Published:** 2024-08-29

**Authors:** Hoda Fouda, Olfat Elsayed Hassanein, Shehabeldin Saber, Mohamed Fouad Haridy, Maha El Baz, Hend Sayed Ahmed, Ahmed Abuelezz

**Affiliations:** 1 Conservative Dentistry Department, Faculty of Dentistry, The British University in Egypt, Cairo, Egypt; 2 Conservative Dentistry Department, Faculty of Dentistry, Cairo University, Giza, Egypt; 3 Endodontic Department, Faculty of Dentistry, The British University in Egypt, Cairo, Egypt; 4 Endodontic Department, Faculty of Dentistry, Misr International University, Cairo, Egypt

**Keywords:** Randomized controlled trial, Root canal treated teeth, Endodontically treated teeth, Non- vital teeth, Inlays, Onlays, Cusp coverage, Cusp preservation, CAD CAM, Composite blocks, Milled composite restorations, Clinical performance, USPHS criteria

## Abstract

**Trial design:**

This is a randomized, controlled, superiority, double-blinded, parallel-group, two-arms trial with an allocation ratio of 1:1. This study aimed to assess whether the cavity design could affect the clinical performance of the CAD/CAM generated indirect resin composite restoration in endodontically treated teeth (ETT) evaluated using the Modified USPHS criteria after a two-year follow up.

**Methods:**

A total of 30 participants who underwent endodontic treatment for MOD cavities in permanent molars were divided randomly into two parallel groups (*n* = 30 restorations) according to the performed cavity design to group 1 in which there was no cuspal reduction (inlay) and group 2 in which cuspal reduction was performed (overlay). All pulp chambers were filled with bulk fill flowable composite, and the cavities were prepared following the criteria of the cavities for indirect restorations and restored using nano-hybrid composite resin blocks (Brilliant, Coltene, Switzerland). The restorations were evaluated using the modified USPHS criteria at baseline, six months, one-year and two years follow-up visits. For qualitative data, frequencies (n) and percentages (%) were used to display the data, while mean and standard deviation (SD) were used for quantitative data. The normality of the data was evaluated using the Shapiro-Wilk and Kolmogorov-Smirnov tests. For every test, *P* ≤ 0.05 was used as the significance threshold.

**Results:**

Twenty-six individuals completed the follow-up period after receiving the assigned intervention.The inter-group comparison showed that, at the 6- months and 12- months observation points, the overlay design had significantly better marginal adaptation, less incidence of discoloration or tooth/restoration fracture, and similar marginal integrity and caries incidence to the inlay design. After 24- months, the overlay design still had better marginal adaptation, less incidence of discoloration or tooth/restoration fracture and less caries incidence in comparison to the inlay design, while there was no difference in the marginal integrity between either design.

**Conclusions and clinical relevance:**

Cuspal reduction in endodontically treated teeth showed better clinical performance than the cusp preservation thus, the former is more reliable.

**Supplementary Information:**

The online version contains supplementary material available at 10.1186/s12903-024-04725-5.

## Introduction

Endodontically treated teeth (ETT) may fail for mechanical or biological causes. Although there are many outcome studies in endodontics that show how successful root canal therapy is, it is well accepted that structural failure is the most frequent cause of ETT loss.[1].

The biomechanical behavior of ETT is affected by the amount of lost tooth structure before, during and after treatment [2, 3], dehydration and changes in collagen fibril cross-linking [4], reduced proprioception and protective reflexes during mastication [5], reduced resilience [6], tooth position and alignment [7], occlusion in terms of magnitude and direction of the functional loads, and equally important, the quality of the coronal restoration [8].

The significance of the final restoration on long-term results has been emphasized by the advent of studies pertaining to the survival and serviceability of ETT rather than absence of clinical and radiographic signs or symptoms of failure. [9]. Therefore, preserving the maximum amount of sound tooth structure, providing a suitable cavity design, and selecting a durable restorative material are considered key factors for longevity of ETT [10–12].

Clinicians often face dilemmas about the best protocol to restore mutilated ETT [13]. Advances in adhesive strategies and CAD/CAM technologies indorsed the use of partial indirect restorations (PIR) to restore mutilated ETT. PIR include inlays (without cuspal coverage), onlays (covering at least 1 cusp), and overlays (covering all cusps) [14]. Numerous resin or ceramic materials are currently available for fabricating PIR.

CAD/CAM composites combine the benefits of ceramics, such as longevity, enamel-like surface quality, anatomic shape, proximal contact, and color stability, with the benefits of composite resin, such as high flexural strength, low abrasiveness, ease of polishing, and repairability [15]. In addition to minimizing the primary drawback of direct composite which is polymerization shrinkage, and limiting it to the luting cement’s thin layer only [16]. Brilliant Crios (Coltène Whaledent,Altstätten ,Switzerland) is a nano-hybrid composite with a multimodal composition of barium glass, and amorphous silica in combination with a reinforcing cross-linked methacrylate matrix [17]. A recent systematic review concluded that indirect resin-based composite restorations are as reliable as PIR, with clinical performance comparable to that of glass-ceramic restorations. [18]. Yet, prospective clinical data on CAD-CAM nano-hybrid composite restorations for ETT are required to validate such assumption. Moreover, the choice of cuspal coverage while restoring mutilated ETT is also controversial and needs more evidence form controlled clinical trials.

Thus, using the modified USPHS criteria, this study aimed to assess the clinical performance of inlays and overlays fabricated from CAD-CAM nano-hybrid composite that were intended to restore badly broken down ETT. The null hypothesis tested was that there should be no difference in the clinical performance of CAD-CAM nano-hybrid composite inlays or overlays after two years of clinical service.

## Materials and methods

### Study setting

The Research Ethics Committee at Cairo University in Egypt (CREC) granted approval for the research design (approval number: 20 9 20, Date: 29/09/2020). Participants were chosen from Cairo University’s Faculty of Dentistry’s outpatient clinic for Conservative Dentistry. The reasons, advantages, dangers, and potential consequences of the therapy were explained to each patient. Each participant completed an informed consent form that was in a written form. The Helsinki Declaration was followed in all procedures carried out for this investigation. On 23/09/2020, the research protocol was registered in the Clinical Trials.gov database with the unique identification number NCT04561167.

### Trial design

The study was a randomized controlled clinical trial, with two parallel groups design, 1:1 allocation ratio and equivalence framework.

### Sample size calculation

A power analysis was designed to have adequate power to apply a statistical test of the null hypothesis that the clinical performance of the indirect resin composite restorations in endodontically treated teeth is similar whether the cavities are prepared with or without cuspal reduction. According to the results of Koyuturk et al.[19]. By adopting an alpha (α) level of 0.05 (5%), and power of (80%). The predicted sample size was 24. In order to account for potential dropouts during the follow-up period, the sample size was expanded by 20%, making a total of [30] cases that is, [15] for each group. G*Power 3.1.9.2, a superiority framework and a chi-square test, were used to calculate the sample size.

### Eligibility criteria

#### Inclusion criteria

Patients included in the study were healthy males and females (Category: American Society of Anesthesiologists class 1, aged 21–45 years, presenting with good oral hygiene, healthy periodontium, a single, endodontically treated mature molar, presence of two remaining walls (MOD) not less than 2 mm thick, clinically and radiographically asymptomatic, and the antagonist teeth are in normal occlusion.

The endodontic treatment was performed at the Endodontic Department at the Faculty of Dentistry, Cairo University using a standardized protocol of cleaning, shaping and obturation.

#### Exclusion criteria

Patients presenting one of the following conditions were excluded from the study: systemic disease (ASA 2–6), pregnancy or breastfeeding, hypersensitivity, uncertain quality of the endodontic treatment, eugenol-based or compromised temporary coronal restoration, multiple teeth treated, patients with parafunctional habits or temporomandibular joint disorders, high caries index, or active periodontal disease.

### Randomization and blinding

**Fig. 1:**
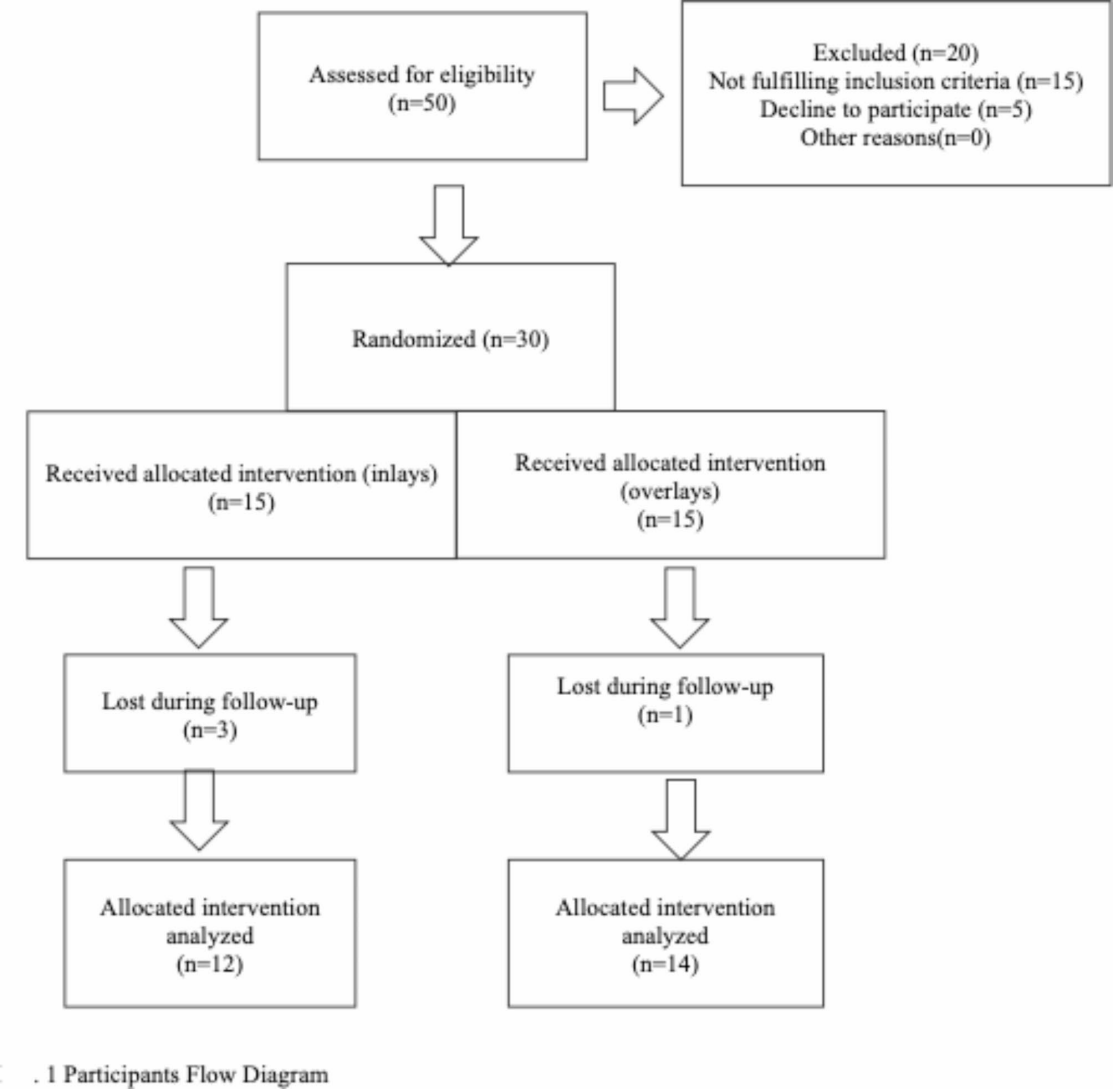
Participants flow diagram

Individuals who met the eligibility criteria were divided into two groups at random with a 1:1 allocation ratio using computer-generated randomization (www.random.org) (15 individuals in each group). MH inserted the successively produced numbers into opaque envelopes up to the intervention time. It was OH who enrolled the participants. To choose their group for the following intervention, each participant was prompted to choose an envelope. Participants were allocated to interventions by HO. Because of the nature of the intervention utilized, blinding the operator and assessors was not possible, however blinding the participants and the statistician were.The participants flow chart shown in Fig. (1).

### Intervention

Demographic data were recorded including each patient’s medical and dental history. Radiographic examination was performed to check for the quality of the endodontic treatment. Clinical examination was performed to confirm absence of any clinical signs or symptoms, to assess the intactness of the temporary coronal restoration, and to assess centric and eccentric occlusal contacts.

### Restorative procedures

The materials used in the study are listed in Table (1). All procedures were performed by the same operator (HF) with more than 10 years of clinical experience, with quadrant rubber dam isolation using 3.5X magnifying loupes (Univet, Italy) with LED light.

**Table 1:** Technical information of the materials used in the clinical study

Material Name	Specifications	Manufacturer	Composition
Brilliant Crios ^®^	Resin Composite Block	Coltène Whaledent,Altstätten, Switzerland	Barium glass Size < 1.0 μm Amorphous Silica SiO2 Size < 20 nm Resin matrix Cross-linked methacrylates Pigments Inorganic pigments such as ferrous oxide or titanium dioxide.
Rely X Unicem Clicker™ Dispenser	Self-Adhesive Universal Resin Cement	(3 M ESPE, St Paul, MN USA)	Base paste (white) Methacrylate monomers containing phosphoric acid groups Methacrylate monomers Silanated fillers Initiator components Stabilizers Catalyst paste (yellow) Methacrylate monomers Alkaline (basic) fillers Silanated fillers Initiator components Stabilizers Pigments
Aluminum OxidePowder	Abrasive powder	Velopex International, UK	Aluminum Oxide Al2O3 powder - particle size 29 μm
Prime&Bond universal™	Universal adhesive	Dentsply Sirona	Bi- and multifunctional acrylate: Surface active crosslinker Phosphoric acid modified acrylate resin: Etchant, adhesion promoter primer Initiator: Photo-initiator system Stabilizer: Stabilize monomers upon storage Isopropanol: Solvent for the resins, polarity adjustment Water: Solvent for the resins, etching aid
Scotchbond™ Universal Etchant	Universal etchant	(3 M ESPE, St Paul, MN USA)	32% phosphoric acid by weight pH approximately 0.1. fumed silica and a water soluble polymer
SureFil™ SDR flow [Smart Dentin Replacement] (Universal Shade)	Visible light cured bulk-fill flowable base resin composite	*DENTSPLY sirona*,* Konstanz*,* Germany*	Matrix: • SDR^™^ patented UDMA resin, • TEGDMA • DMA resin, • Di-functional diluents. • EBPADMA • triethyleneglycol Di methacrylate Filler: • Barium and Strontium. • Fluoroalumino-silicate glasses. (68% by wt., 45% by vol.)

### Cavity preparation

After temporary filling removal, the cavity was checked for any remaining caries that was removed using sharp hand excavators. All undermined enamel was removed. Any gutta-percha remnants in the pulp chamber were removed till the level of the root canal orifice. Standardization of the prepared cavity dimensions was done as follows [20]:

1. In the inlay group, thickness of the remaining walls were ≥ 2 mm measured by a dental caliper.
2. In the overly group, cuspal reduction was performed for the buccal and lingual walls so that the occlusal intercuspal distance ranged from 4 to 5 mm in maximum intercuspation and during lateral movements. measured by a graduated periodontal probe .
3. Using the same diamond bur (Komet, USA) used for the occlusal part, the buccal and lingual walls of the proximal portions of the cavity were prepared to produce the same angle of divergence (6 degrees).
4. The pulpal floor of the occlusal portion of the cavity and the gingival floor of the proximal part of the cavity were prepared to be continuous at the same depth.
5. The cavosurface angles were 90° and the internal line angles were rounded.
6. All cavities were optimized by restoring the pulp chamber till the level of the pulpal floor using Bulk fill flowable composite (**SureFil™** SDR, Dentsply Sirona).

### Restoration construction

To take an optical impression, each prepared tooth was scanned using the Omnicam intraoral camera of the CEREC system software version 4.60 (Sirona Dental Systems GmbH, D-64,625 Benshein, Germany) [21]. The margin was drawn and the final design was obtained and verified using the CEREC software version 4.60. In order to machine composite restorations, the occlusal and lateral wall thickness parameters were set to 1.5 mm and 100 μm, respectively, for the cement space. Using the MCXL milling machine (Sirona, USA), size 14 nanohybrid CAD/CAM composite blocks (Brilliant blocs) were used to manufacture the indirect restorations.

### Cementation protocol

The fitting surface of the restoration was cleaned and roughened with 29 microns of aluminum oxide using an intraoral sandblaster equipment (Aquacare, Velopex, UK). [22]. The restoration was then placed in an ultrasonic cleaner filled with distilled water and left for four minutes. [23]. After being taken out of the cleaner, the fitting surface was allowed to air dry gently. Prime and Bond universal adhesive (Dentsply Sirona) was applied and actively rubbed for 20 s, solvent evaporation was allowed for 20 s before light-curing for 10 s using LED curing light (Elipar S10, 3 M ESPE) at a light intensity of 1200 mw/cm2. Then, 35% phosphoric acid gel (Scotchbond™ Universal Etchant 3 M) was applied to the enamel margins for 15 s, then rinsed for 30 s and gently air dried. After 20 s of active application, 5 s of gentle air drying, the adhesive was light cured for 10 s. Using the auto-mix tip supplied by the manufacturer, a dual-cured adhesive resin cement (RelyX Unicem clicker 3 M ESPE) was injected into the cavity. After that, the restoration was inserted into the cavity and its entire seating was verified. To make it easier to remove the interproximal and marginal excess using dental floss, the cement was first light-cured for two seconds. To obtain the final set, light curing was then performed for forty seconds in each direction.

### Contact, occlusal checking and finishing and polishing

Using an unwaxed dental floss (Oral-B, USA), the proximal contacts were examined. An articulating paper (Blue Red Combo 0.0028”/71 µm, Crosstex ^®^ International, USA) was used to adjust the occlusal contacts. Lastly, polishing was carried out with rubber points (Enhance kit, Dentsply Sirona) operating at low speed contra-angle handpiece (NAC-EC, NSK, Japan) with a maximum speed of 20,000 rpm under water coolant and minimal pressure. Finishing was completed using fine grit yellow coded tapered with round and flame diamond stones (#368EF, #852EF, Komet, USA).

### Outcome assessment

Mechanical and biological assessment of the dental restoration was done in adherence to the modified USPHS criteria via clinical and radiographic examination according to Table (2) by 2 assessors (OH and MH), with more than 15 years clinical experience, to evaluate the restorations post-cementation and after 6,12, and 24 months. In the event of differences, a consensus was reached after discussion with a third assessor (SS).

**Table 2:** Modified USPHS criteria, score, characteristics, measuring unit and method of diagnosis for assessment of dental restorations

Outcome	Method of evaluation	Unit of measurement
Marginal adaptation	Clinical	Alpha: Closely adapted, no visible crevice.
		Bravo: Visible crevice, explorer will penetrate.
		Charlie: Crevice in which dentin is exposed.
Marginal Integrity	Clinical	Alpha: sound.
		Bravo: Positive Step (could be removed by finishing)
		Charlie: slight negative step, not removable, localized.
		Delta: strong negative step in major parts of the margin, not removable.
Marginal Discoloration	Clinical	Alpha: No discoloration.
		Bravo: Discoloration without penetration in pulpal direction.
		Charlie: Discoloration with penetration in pulpal direction.
Fracture	Clinical	Alpha: restoration retained, no fractures of cracks
		Bravo: hairline cracks and/or chipping not affecting the marginal integrity or proximal contacts
		Charlie: partial or complete loss of restoration
Secondary Caries	Clinical and Radiographical	Alpha: No caries presents
		Charlie: Caries presents.

### Statistical analysis

IBM SPSS Statistics Version 2.1 for Windows was used for statistical analysis. For qualitative data, the presentation was in frequencies (n) and percentages (%), whereas for quantitative data, the presentation methods were mean and standard deviation (SD). The normality of the data was evaluated using the Shapiro-Wilk and Kolmogorov-Smirnov tests. A significant threshold of *P* ≤ 0.05 was established. Chi-square test was applied to intergroup comparisons of qualitative data pertaining to demographic data. Since the participants’ ages demonstrated a normal distribution, the Independent Student-t test was employed to compare groups. The frequency of modified USPHS scores of each outcome between the two cavity designs at each evaluation period was compared using the chi-square test. Cochran Q test was used to compare the frequency of modified USPHS scores of each outcome between different evaluation times within each cavity design. Inter-evaluator reliability was analyzed using Kappa Test.

## Results

Intergroup comparisons for demographic data showed no significant differences between both groups regarding sex (*p* = 0.713), age (*p* = 0.539), arch (*p* = 0.439) or tooth type (*p* = 0.690). Recruitment ceased on March 31, 2021, following the enrollment of the target population, on January 12, 2020. Follow up began on January 12, 2022, and finished on March 31, 2023. The designated intervention was given to every participant. After completing the analysis, 26 individuals had their results examined. During the follow-up period, three individuals from the inlay group and one person from the overlay group were absent. The flow diagram for participants is shown in Fig. 1.The Kappa score of the inter-evaluator reliability was 0.97. Tables (3, 4, 5, 6 and 7) provide the result score frequencies and percentages for both groups at each observation point. The inter-group comparison showed that, at the 6- months and 12- months observation points, the Onlay design had significantly better marginal adaptation, less incidence of discoloration or tooth/restoration fracture (*p* < 0.05), and similar marginal integrity and caries incidence (*p* = 1) to the inlay design. After 24- months, the Onlay design still had better marginal adaptation, less incidence of discoloration or tooth/restoration fracture and less caries incidence (*p* < 0.05) in comparison to the inlay design, while there was no difference in the marginal integrity between either design (*p* = 1).

**Table 3:** Frequency distribution of the USPHS criteria scores for marginal adaptation of both cavity designs at each evaluation time

Outcome	Evaluation time	Inlay				Overlay			*P*-value
		Alpha	Bravo	Charlie		Alpha	Bravo	Charlie	
Marginal adaptation	Baseline (*n* = 15)	15 (100%) ^A^	0 (0%)^B^	0 (0%) ^B^	Baseline (*n* = 15)	15 (100%)^A^	0 (0%) ^B^	0 (0%)^A^	1.000NS
	6 months (*n* = 14)	7 (50%)^Bb^	7 (50%)^Aa^	0 (0%) ^B^	6 months (*n* = 14)	14 (100%)^Aa^	0 (0%) ^Bb^	0 (0%)^A^	0.002*
	12 months (*n* = 12)	4 (33.3%)^Bb^	8 (66.7%)^Aa^	0 (0%) ^B^	12 months (*n* = 14)	14 (100%)^Aa^	0 (0%) ^Bb^	0 (0%)^A^	< 0.001*
	24 months (*n* = 12)	2 (16.7%)^Bb^	5 (41.7%)^Aa^	5 (41.7%) ^Aa^	24 months (*n* = 14)	11 (78.6%)^Aa^	3 (21.4%)^Aa^	0 (0%)^Ab^	0.003*
	P-value	< 0.001*				0.029*			

*: significant at *P* ≤ 0.05; NS: non-significant at *P* > 0.05

Different lowercase superscript letter denotes a significant difference in the proportions of the same score between cavity designs at *P* ≤ 0.05

Different uppercase superscript letter denotes a significant difference in the proportions of the same score between evaluation times at *P* ≤ 0.05

**Table 4:** Frequency distribution of the USPHS criteria scores for marginal integrity of both cavity designs at each evaluation time

Outcome	Evaluation time	Inlay				Overlay			*P*-value
		Alpha	Bravo	Charlie		Alpha	Bravo	Charlie	
Marginal integrity	Baseline (*n* = 15)	15 (100%) ^A^	0 (0%) ^B^	0 (0%) ^A^	Baseline (*n* = 15)	15 (100%) ^A^	0 (0%) ^B^	0 (0%) ^A^	1.000NS
	6 months (*n* = 14)	14 (100%) ^A^	0 (0%) ^B^	0 (0%) ^A^	6 months (*n* = 14)	14 (100%) ^A^	0 (0%) ^B^	0 (0%) ^A^	1.000NS
	12 months (*n* = 12)	14 (100%) ^A^	0 (0%) ^B^	0 (0%) ^A^	12 months (*n* = 14)	14 (100%) ^A^	0 (0%) ^B^	0 (0%) ^A^	1.000NS
	24 months (*n* = 12)	11 (78.6%) ^A^	3 (21.4%) ^A^	0 (0%) ^A^	24 months (*n* = 14)	11 (78.6%) ^A^	3 (21.4%) ^A^	0 (0%) ^A^	1.000NS
	P-value	0.021*				0.029*			

*: significant at *P* ≤ 0.05; NS: non-significant at *P* > 0.05

Different uppercase superscript letter denotes a significant difference in the proportions of the same score between evaluation times at *P* ≤ 0.05

**Table 5:** Frequency distribution of the USPHS criteria scores for marginal discoloration of both cavity designs at each evaluation time

**Outcome**	Evaluation time	Inlay				Overlay			P-value
		Alpha	Bravo	Charlie		Alpha	Bravo	Charlie	
**marginal discoloration**	**Baseline (n = 15)**	15 (100%) ^A^	0 (0%) ^B^	0 (0%) ^A^	**Baseline (n = 15)**	15 (100%)	0 (0%)	0 (0%)	1.000NS
	**6 months (n = 14)**	7 (50%) ^Bb^	7 (50%) ^Aa^	0 (0%) ^A^	**6 months (n = 14)**	14 (100%)^a^	0 (0%)^b^	0 (0%)	0.002*
	**12 months (n = 12)**	5 (41.7%) ^Bb^	7 (58.3%) ^Aa^	0 (0%) ^A^	**12 months (n = 14)**	14 (100%)^a^	0 (0%)^b^	0 (0%)	0.001*
	**24 months (n = 12)**	2 (16.7%) ^Bb^	10 (83.3%) ^Aa^	0 (0%) ^A^	**24 months (n = 14)**	14 (100%)^a^	0 (0%)^b^	0 (0%)	< 0.001*
	**P-value**	< 0.001*				1.000NS			

*: significant at *P* ≤ 0.05; NS: non-significant at *P* > 0.05

Different lowercase superscript letter denotes a significant difference in the proportions of the same score between cavity designs at *P* ≤ 0.05

Different uppercase superscript letter denotes a significant difference in the proportions of the same score between evaluation times at *P* ≤ 0.05

**Table 6:** Frequency distribution of the USPHS criteria scores for fracture of tooth/restoration of both cavity designs at each evaluation time

Outcome	Evaluation time	Inlay				Overlay			*P*-value
		Alpha	Bravo	Charlie		Alpha	Bravo	Charlie	
Fracture of tooth/restoration	Baseline (*n* = 15)	15 (100%)^A^	0 (0%)^B^	0 (0%)^B^	Baseline (*n* = 15)	15 (100%) ^A^	0 (0%)^B^	0 (0%) ^A^	1.000NS
	6 months (*n* = 14)	7 (50%)^Bb^	7 (50%) ^Aa^	0 (0%)^B^	6 months (*n* = 14)	14 (100%) ^Aa^	0 (0%)^Bb^	0 (0%) ^A^	0.002*
	12 months (*n* = 12)	4 (33.3%)^Bb^	8 (66.7%)^Aa^	0 (0%)^B^	12 months (*n* = 14)	14 (100%) ^Aa^	0 (0%)^Bb^	0 (0%) ^A^	< 0.001*
	24 months (*n* = 12)	2 (16.7%)^Bb^	5 (41.7%) ^Aa^	5 (41.7%) ^Aa^	24 months (*n* = 14)	11 (78.6%) ^Aa^	3 (21.4%) ^Aa^	0 (0%) ^Ab^	0.003*
	P-value	< 0.001*				0.029*			

*: significant at *P* ≤ 0.05; NS: non-significant at *P* > 0.05

Different lowercase superscript letter denotes a significant difference in the proportions of the same score between cavity designs at *P* ≤ 0.05

Different uppercase superscript letter denotes a significant difference in the proportions of the same score between evaluation times at *P* ≤ 0.05

**Table 7:** Frequency distribution of the USPHS criteria scores for secondary caries of both cavity designs at each evaluation

Outcome	Evaluation time	Inlay			Overlay		*P*-value
		Alpha	Charlie		Alpha	Charlie	
Secondary caries	Baseline (*n* = 15)	15 (100%) ^A^	0 (0%)^B^	Baseline (*n* = 15)	15 (100%)	0 (0%)	1.000NS
	6 months (*n* = 14)	14 (100%) ^A^	0 (0%)^B^	6 months (*n* = 14)	14 (100%)	0 (0%)	1.000NS
	12 months (*n* = 12)	12 (100%) ^A^	0 (0%)^B^	12 months (*n* = 14)	14 (100%)	0 (0%)	1.000NS
	24 months (*n* = 12)	2 (16.7%)^Bb^	10 (83.3%) ^Aa^	24 months (*n* = 14)	13 (92.9%)^a^	1 (7.1%)^b^	< 0.001*
	P-value	< 0.001*			0.392NS		

*: significant at *P* ≤ 0.05; NS: non-significant at *P* > 0.05

Different lowercase superscript letter denotes a significant difference in the proportions of the same score between cavity designs at *P* ≤ 0.05

Different uppercase superscript letter denotes a significant difference in the proportions of the same score between evaluation times at *P* ≤ 0.05

## Discussion

A recent systematic review and meta-analysis for the influence of PIR on the clinical prognosis of ETT revealed that the failure rate of PIR after 2–4 years of clinical service was 4.32%. This increased to 10.65% after 7 years, and to 20.94% after 12–30 years, with most failures appearing restorable [24]. Although such results validate PIR to restore mutilated ETT, yet the choice of the best PIR design needs more clinical evidence from randomized controlled trials. This study compared the clinical performance of inlays versus overlays for restoring ETT. Clinical cases with MOD extensions were chosen because they are associated with high cuspal deflection [25] in addition to a marked decrease in tooth stiffness [26] and fatigue life [27].The inlay design, with cusp preservation, was chosen being reported to reduce cusp deflection and increase the strength of the remaining dental structure [28]. To lessen bias during selection of the participants, a clear and comprehensive screening criteria was established, followed by developing a transparent recruitment protocol. Patients who were less cooperative or unwilling to participate were offered the treatment planned regardless from their wish to non-participate in the trial. Finally, the reasons for non-participation were analyzed, which was principally the fear of commitment to maintain the long follow-up period. The overlay design, with cusp coverage, was chosen being reported to preserve tooth contours, resulting in more proper function as well as significantly preserving the tooth structure when compared with full crowns [29] For the inlay group, a minimum thickness of cusps of 2 mm was mandatory to allow for cusp preservation otherwise, the recruited patient was either excluded from the study or was converted to the other group of cuspal reduction with replacement. Regarding overlay group, cusp reduction was done flat to provide a butt joint for optimal analysis of the occlusal forces along the remaining axial walls [30].

It is widely accepted that the USPHS criteria, which have been in use for defining clinical outcomes throughout time, are dependable and conventional. [31]. Though, other studies suggest that the USPHS criteria has a limited sensitivity compared to the FDI criteria [32]. One major benefit of CAD-CAM restorations is that they may be finished quickly following endodontic therapy, often even on the same visit. There is insufficient data on endodontic- restorative- therapy performed in a single visit. Retrospective data point out that prompt indirect restoration yields superior results. [1]. Therefore, it was decided to restore teeth that were endodontically treated within a maximum of two weeks.

A variety of tactics were used for reducing the stress concentration at the bonding interface. This involved filling the pulp chamber up to the pulpal floor level with SDR flowable composite, which has a shrinkage stress modulator. According to reports, this method can reduce gap development, microleakage, and secondary caries over time as well as the polymerization stresses produced at the dentine-bond interface.[1]. Additionally, smoother dentine walls with rounded internal line angles are produced by air-abrasion with aluminum oxide priming dentine before restorative procedures. This is said to reduce stress concentration along the bonding interface because of a lower C-factor.[1].

According to the results of this study, the null hypothesis has to be rejected. After 2 years of clinical service, the overlay design had better marginal adaptation, less incidence of caries, less marginal discoloration, and less incidence of tooth/restoration fracture (*p* < 0.05) in comparison to the inlay design, while there was no difference in the marginal integrity between either design (*p* = 1). Inferior marginal adaptation of inlays in previous studies have been attributed to the pattern of stress analysis at the tooth-restoration interface. Dejac and Mlotkowski in 2020 [34], using finite element analysis, examined the mvM stresses in inlays, onlays, and endocrowns constructed of various materials and their bonding with molars. They came to the conclusion that teeth with inlay restorations had the highest values and adverse stress levels. Moreover, they recommended that MOD cavities in molars should be reconstructed with cusp-covering restorations. Results of our study agrees with Chrepa et al. in 2014 [35] who retrospectively evaluated composite onlays on ETT after 37 months of clinical service and reported that 97.4% of the restorations were rated with alpha scores regarding occlusal and proximal marginal adaptation, which was attributed to the low elastic modulus of the composite as well as the protective cuspal-coverage design of the onlays.

Results of this study revealed a significantly higher incidence of caries and marginal discoloration with inlays at the end of the observation period. This can be also attributed to analysis of the functional stresses at the tooth-restoration interface resulting in deterioration of the luting cement, followed by marginal leakage, roughness, and stain retention. [36–38]

Regarding restoration/tooth fracture, the current study identified significantly lower incidence in alpha scores for inlays at all observation periods. This can be explained by the difference in wear rate between enamel and composite, resulting in making the composite more vulnerable to chipping during functional loading [39]. Conversely, it has been reported that when overlays are subjected to occlusal load, the maximum stress occur in the center of the occlusal surface rather than at the margins of the restoration [34]. Our findings agree with Chrepa et al. in (2014) [35] who described composite onlays as a viable option with 100% tooth survival and 96.8% restoration survival rates after 37 months of clinical service. More weight should be placed in future research on the kinds of failures assessed in clinical studies than on the failure rate per se. It is essential to distinguish between irreversible failures, which lead to tooth extraction, and reversible ones, which enable the clinician to replace or repair the restorative [40]. Nowadays, this factor is vital since it changes the meaning of failure to a more conservative and distinct concept, emphasizing tooth preservation above all else rather than the restoration’s long-term stability in the oral cavity. Future studies can also address the impact of minimally invasive endodontic access designs on the survival rate of ETT restored with inlays or overlays.

Marginal integrity, on the other hand, was comparable between inlays and overlays at all observation periods. This can be explained on basis of strict adherence to the principles of cavity preparation, restoration construction, bonding, cementation, finishing and polishing.

The age range selected in this study was mainly to ensure that we only restore primary carious lesions. This would avoid more tooth destruction, or the presence of dentin cracks associated with old restorations. In addition, this age range made patient-retention more pertinent for the two years follow-up period. We acknowledge that our findings are primarily applicable to the (13–18 years) age group and may not fully represent the biomechanical responses of endodontically treated molars in older adult populations. Therefore, we recommend further research to investigate the biomechanical behavior of endodontically treated molars in a broader age range with subgroup analysis to enhance the external validity of the study.

Due to the non-split-mouth design, another limitation of this trial is the absence of occlusal load homogeneity. However, patients with worn facets and parafunctional behaviors, were not allowed to participate in the study. It is also recommended to extend the follow-up period in order to assess the outcomes measured. Considering the challenges in restoring ETT with MOD extensions, cusp protection with indirect composite overlays is strongly recommended over inlays. Still, extended follow-up periods are recommended to to assess long-term outcomes related to cuspal reduction.

### Electronic supplementary material

Below is the link to the electronic supplementary material.

Supplementary Material 1

Supplementary Material 2

Supplementary Material 3

Supplementary Material 4

Supplementary Material 5

Supplementary Material 6

## Data Availability

data is provided within the manuscript and as supplementary information files.
