# Supplementary material for: Two-year clinical performance of indirect resin composite restorations in endodontically treated teeth with different cavity preparation designs: a randomized clinical trial
Source: BMC Oral Health. 2024 Aug 29;24:1009. doi: 10.1186/s12903-024-04725-5 (PMC11363545; doi:10.1186/s12903-024-04725-5)
Supplement: Supplementary file 1 — Supplementary Material 1 [file 12903_2024_4725_MOESM1_ESM.pdf]

Cairo University
Faculty of Dentistry
Research Ethics Committee

جامعة القاهرة
كلية طب الأسنان
لجنة أخلاقيات البحث العلمي

# موافقة

تمت الموافقة على إجراء البحث العلمي المقدم من الطبيبة / هدى عمر توفيق فودة المسجلة بدرجة الدكتوراه بقسم العلاج التحفظي وعنوان البحث/

***Clinical Performance of the Indirect Resin Composite Restorations in Endodontically Treated Teeth with Different Cavity Preparation Designs . (A randomized clinical trial).***

وذلك بعد فحصه من قبل لجنة اخلاقيات البحث العلمي في جلسته بتاريخ ٢٠٢٠/٩/٢٩بعد التأكد من أن البحث يتوافق مع الميثاق الاخلاقى للبحث العلمي ، وأن الفائدة المرجوة منه تربو على المخاطر المحتملة.

| | | |
|---|---|---|
| 20 | 9 | 20 |

رئيس اللجنة

[illegible]

أ.د/ إيمان محمد انور

[illegible]
١/٤

11 Elsaraya Street , Manial , Cairo, Egypt
Tel.( 002) (02) 23642705/ 23642483/ Ext 522
Fax( 002) (02) 23646375

١١ شارع السرايا - المنيل - القاهرة
ت: ٢٣٦٤٢٧٠٥ - ٢٣٦٤٢٤٨٣ - داخلى ( ٥٢٢ )
فاكس: ٢٣٦٤٦٣٧٥
