# Supplementary material for: Two-year clinical performance of indirect resin composite restorations in endodontically treated teeth with different cavity preparation designs: a randomized clinical trial
Source: BMC Oral Health. 2024 Aug 29;24:1009. doi: 10.1186/s12903-024-04725-5 (PMC11363545; doi:10.1186/s12903-024-04725-5)
Supplement: Supplementary file 4 — Supplementary Material 4 [file 12903_2024_4725_MOESM4_ESM.docx]

Marginal Integrity

| **Patient code** | **baseline** | **6 months** | **12 months** | **2 years** |
| --- | --- | --- | --- | --- |
|  | **score** | **score** | **score** | **score** |
| **In 1** | Alpha | Alpha | Alpha | Alpha |
| **In 2** | Alpha | Alpha | Charlie | Charlie |
| **In 3** | Alpha | Alpha | Alpha | Charlie |
| **In 4** | Alpha | Bravo | Charlie | Delta |
| **In 5** | Alpha | Bravo | Charlie | Charlie |
| **In 6** | Alpha | Alpha | Alpha | Charlie |
| **In 7** | Alpha | Bravo | Charlie | Delta |
| **In 8** | Alpha | Bravo | Charlie | Charlie |
| **In 9** | Alpha | Bravo | Charlie | Charlie |
| **In 10** | Alpha | Bravo | Charlie | Delta |
| **In 11** | Alpha | Bravo | Bravo | Bravo |
| **In 12** | Alpha | Alpha | Alpha | Alpha |
| **In 13** | Alpha | Alpha | Drop out | Drop out |
| **In 14** | Alpha | Alpha | Drop out | Drop out |
| **In 15** | Alpha | Drop out | Drop out | Drop out |

**Key: In; inlay without cusp reduction**

**On; onlay with cusp reduction**

| **Patient code** | **baseline** | **6 months** | **12 months** | **2 years** |
| --- | --- | --- | --- | --- |
|  | **score** | **score** | **score** | **score** |
| **On 1** | Alpha | Alpha | Alpha | Alpha |
| **On 2** | Alpha | Alpha | Alpha | Bravo |
| **On 3** | Alpha | Alpha | Alpha | Bravo |
| **On 4** | Alpha | Alpha | Alpha | Alpha |
| **On 5** | Alpha | Alpha | Alpha | Alpha |
| **On 6** | Alpha | Alpha | Alpha | Alpha |
| **On 7** | Alpha | Alpha | Alpha | Alpha |
| **On 8** | Alpha | Alpha | Alpha | Alpha |
| **On 9** | Alpha | Alpha | Alpha | Alpha |
| **On 10** | Alpha | Alpha | Alpha | Bravo |
| **On 11** | Alpha | Alpha | Alpha | Alpha |
| **On 12** | Alpha | Alpha | Alpha | Alpha |
| **On 13** | Alpha | Alpha | Alpha | Alpha |
| **On 14** | Alpha | Alpha | Alpha | Alpha |
| **On 15** | Alpha | Drop out | Drop out | Drop out |
